# Port site hernia at the robotic arm port after robotic‐assisted laparoscopic radical prostatectomy

**DOI:** 10.1002/iju5.12180

**Published:** 2020-06-14

**Authors:** Taiki Ogasa, Masayoshi Nagata, Hiroki Koyasu, Toshiyuki China, Kosuke Kitamura, Yoshiaki Wakumoto, Satoru Muto, Shigeo Horie

**Affiliations:** ^1^ Department of Urology Juntendo University Graduate School of Medicine Tokyo Japan

**Keywords:** port site hernia, prostatectomy, RARP, robotic surgery, trocar site hernia

## Abstract

**Introduction:**

Complications unique to laparoscopic surgery have been reported, including port site hernia. We experienced a case of port site hernia in the robotic right‐hand port site measuring 8 mm in diameter after robotic‐assisted laparoscopic radical prostatectomy.

**Case presentation:**

A 56‐year‐old man was indicated a high prostate–specific antigen level of 37.8 ng/ml. Subsequent prostate biopsy revealed adenocarcinoma with a Gleason score of 4 + 4. The patient underwent robotic‐assisted laparoscopic radical prostatectomy in Juntendo Hospital. Although his postoperative recovery was generally good, the patient complained of sudden nausea and acute abdominal pain. A contrast computed tomography scan showed an ileus due to a hernia occurring at the robotic right‐hand port, the da Vinci Arm I port. We released incarceration under laparoscopic procedure.

**Conclusion:**

Since the port diameter is relatively small in robot‐assisted surgery, port site hernias are expected to be rare. However, careful attention should be paid to the positional deviation of the remote center.

**Abbreviations & Acronyms**
BMI: body mass index
CT: computed tomography
MRI: magnetic resonance imaging
PSA: prostate‐specific antigen
RARP: robotic‐assisted laparoscopic radical prostatectomy
T2WI: T2‐weighted image

**Keynote message**

In robot‐assisted surgery, the abdominal wall may be loaded unexpectedly due to the displacement of the remote center. Caution is required as this may cause port site hernia.

## Introduction

RARP using the da Vinci surgical system was initially performed in 2000^1^ and has rapidly become a popular surgical technique for prostate cancer. One of the complications in laparoscopic surgery is port site hernia, in which the intestine is incarcerated at the port and causes obstructive ileus. However, reports on port site hernia in robot‐assisted laparoscopic surgery have been rare.^2^ Here, we report a case of post‐RARP port site hernia in the robotic 8‐mm port which required surgical reduction.

## Case presentation

The patient was a 56‐year‐old male with no medical history. His BMI was 25.7 kg/m^2^. Serum PSA level before prostate biopsy was 37.8 ng/ml and MRI showed that prostate cancer with T2WI low intensity and strong diffusion restriction was detected on the left lobe. Moreover, extracapsular invasion suspected. The PI‐RADS (version 2) score was 5.

Pathological findings of the prostate biopsy revealed adenocarcinoma. There was no increase in inflammatory response or anemia, and no other obvious abnormality was observed.

**Fig. 1:**
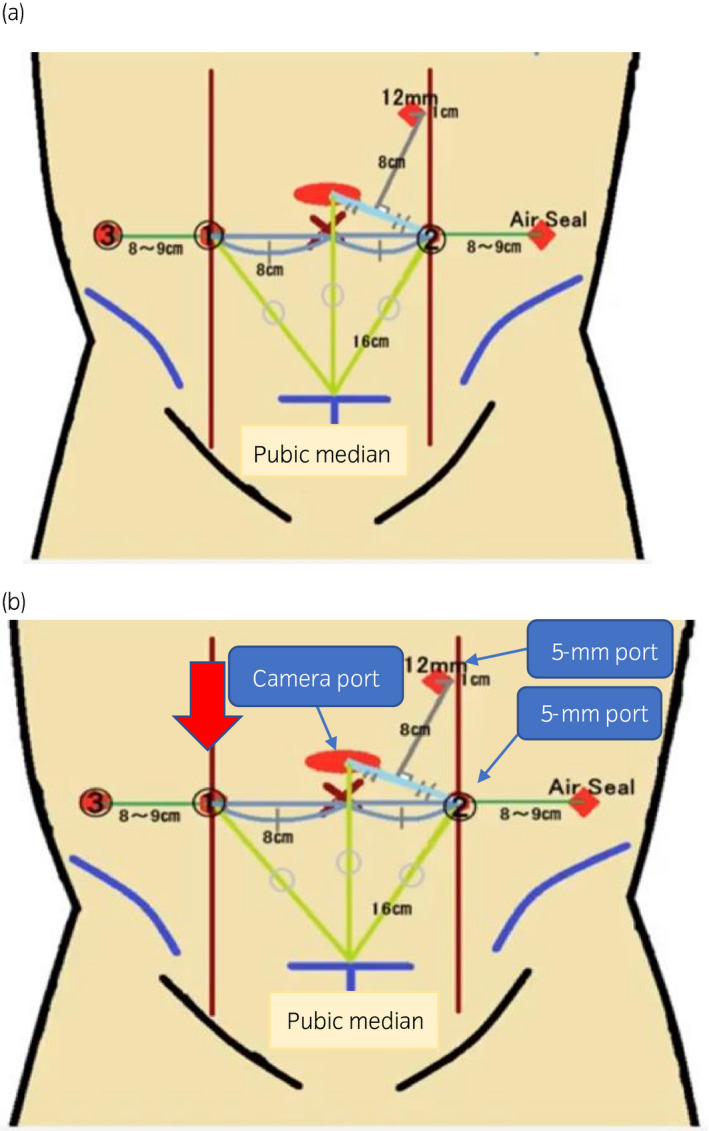
Location of trocars. (a) RARP. A skin incision was placed 16 cm from the midline of the pubic bone and a 12‐mm camera port was placed. Two 8‐mm ports, which are the robotic right‐hand arm and third arm, were created in the right flank. Another 8‐mm port, which is the left‐hand arm of the robot, a 5‐mm port for the left‐hand forceps of the assistant, and a 12‐mm port for the right‐hand forceps of the assistant were disposed on the left upper abdomen. (b) Hernia release surgery. The 5‐mm ports were made at each of the assistant right‐hand forceps port and the robot left‐hand arm port used in the previous RARP. The red arrow indicates the incarceration of the port site hernia.

We performed RARP under diagnosis of adenocarcinoma with cT3aN0M0. A 3.5‐cm skin incision was placed 16 cm from the midline of the pubic bone, a 12‐mm camera port was placed, and 10 mmHg was insufflated (Fig. 1a). Two 8‐mm ports, which were the robotic right‐hand arm and third arm, were created in the right flank. Another 8‐mm port, which was the robotic left‐hand arm, a 5‐mm port for the left‐hand of the assistant, and a 12‐mm port for the right‐hand of the assistant were disposed on the left upper abdomen. The wound was sutured with a 2‐0 absorption thread together with the fascia and peritoneum with one needle. That closure had not been confirmed visually from the abdominal cavity that the fascia and peritoneum were securely sutured. The drain was placed at the robotic third‐arm port. Surgical time was 140 min, console time was 75 min, and bleeding volume was 250 mL.

Pathological findings were adenocarcinoma with Gleason score of 4 + 4, EPE0, RM0, sv0, n0, pT2cN0Mx.

**Fig. 2:**
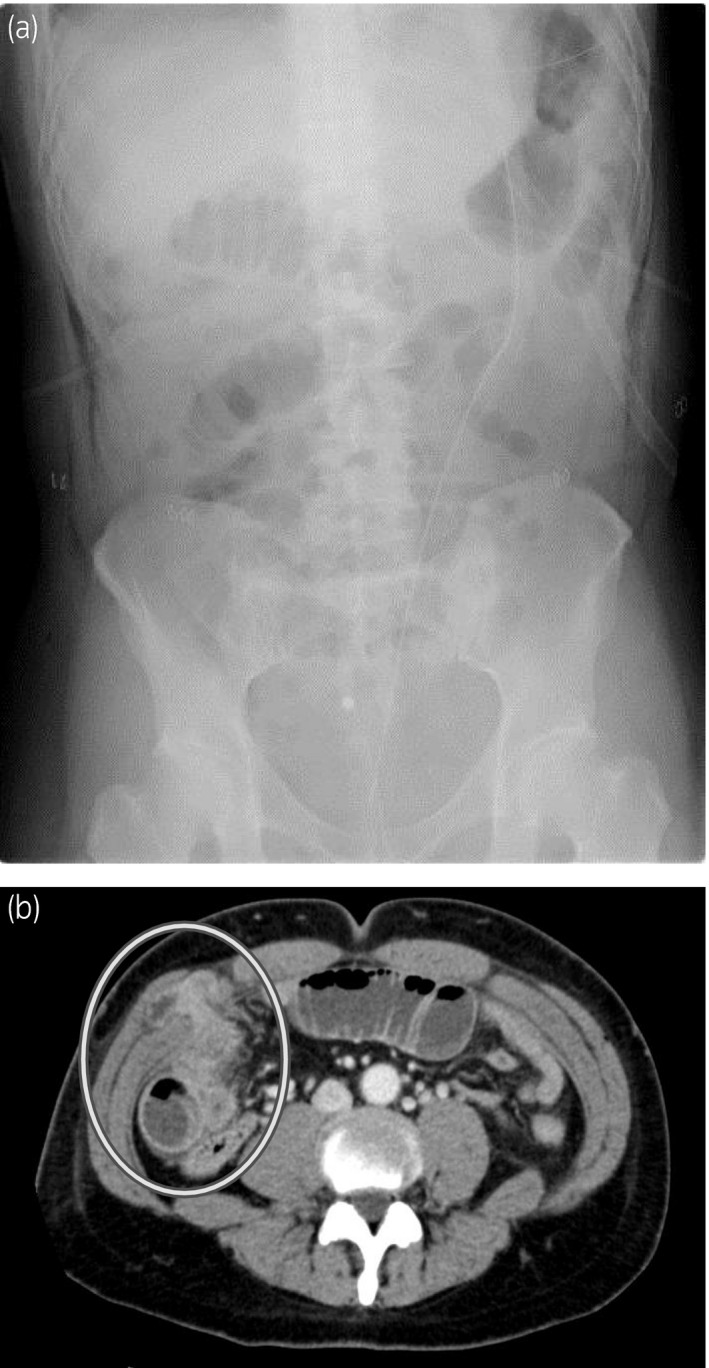
(a) KUB at the onset of hernia. The niveau sign was on the right side of the abdomen, suggesting intestinal obstruction, so we proceeded with a contrast‐enhanced CT scan to detect the cause. (b) Abdominal enhanced CT scan at the onset of hernia. The intestine had escaped from the peritoneum from the port site of the robotic right‐hand arm and the intestine on the oral side was expanding, which was diagnosed as a postoperative ileus due to port site hernia.

**Fig. 3:**
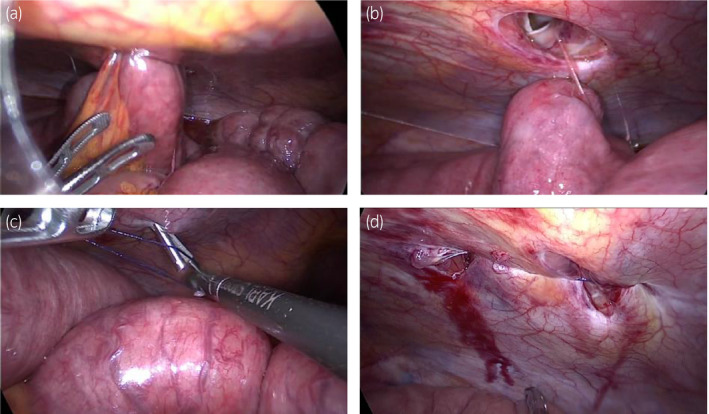
Intraoperative image findings of the hernia repair surgery. (a) Shows inside the abdominal cavity and confirmation of the Richter‐type incarcerated small intestine at the port site of the robotic right‐hand arm. (b) When the incarcerated small intestine was pulled by the grasping forceps, the indwelling was easily released. (c) The fascia and peritoneum were sutured using three endoscopic needles. (d) The port site of the hernia was absolutely closed.

The drain was removed on the first postoperative day. A bowel movement occurred on the second postoperative day, after which a dysphagia advanced diet was normally initiated. The patient suddenly complained of nausea and acute abdominal pain and an abdominal X‐ray showed abnormal intestinal gas on the fourth postoperative day (Fig. 2a). A CT scan showed that the intestine had escaped from the peritoneum from the 8‐mm port site of the robotic right‐hand arm, which was diagnosed as postoperative ileus due to port site hernia (Fig. 2b). As an attempt at manual reduction failed, we decided to perform emergency surgery to release the hernia. We made a camera port at the same site used in the RARP. The 5‐mm ports were made at the assistant’s right‐hand port as well as at the robotic left‐hand arm port used in the RARP (Fig. 1b). We observed the interior of the abdominal cavity and confirmed the Richter‐type incarcerated small intestine at the robotic right‐hand arm port (Fig. 3a). The indwelling was easily released when pulled by the grasping forceps (Fig. 3b). We found no damage or necrosis in the intestinal tract and considered that it could be preserved safely. The fascia and peritoneum were sutured (Fig. 3c) with three endoscopic needles and was then closed (Fig. 3d). The patient was discharged after 14 days of hospitalization.

## Discussion

The incidence of port site hernia after laparoscopic surgery is approximately 0.5%^3^, ^4^ and is reported to be approximately 0.4–0.66% following urological laparoscopic surgery, which is considered to be the same frequency. The overall incidence of port site hernia inclusive of laparoscopy and robot‐assisted surgeries ranges from 0 to 5.2%.^5^

According to a systematic review, risk factors of port site hernia after laparoscopic surgery were age of 60 or more years, BMI of 28 or greater, triangular pyramid port of 12 mm or greater in a port diameter, and more than 80 min of surgical time.^6^

**Fig. 4:**
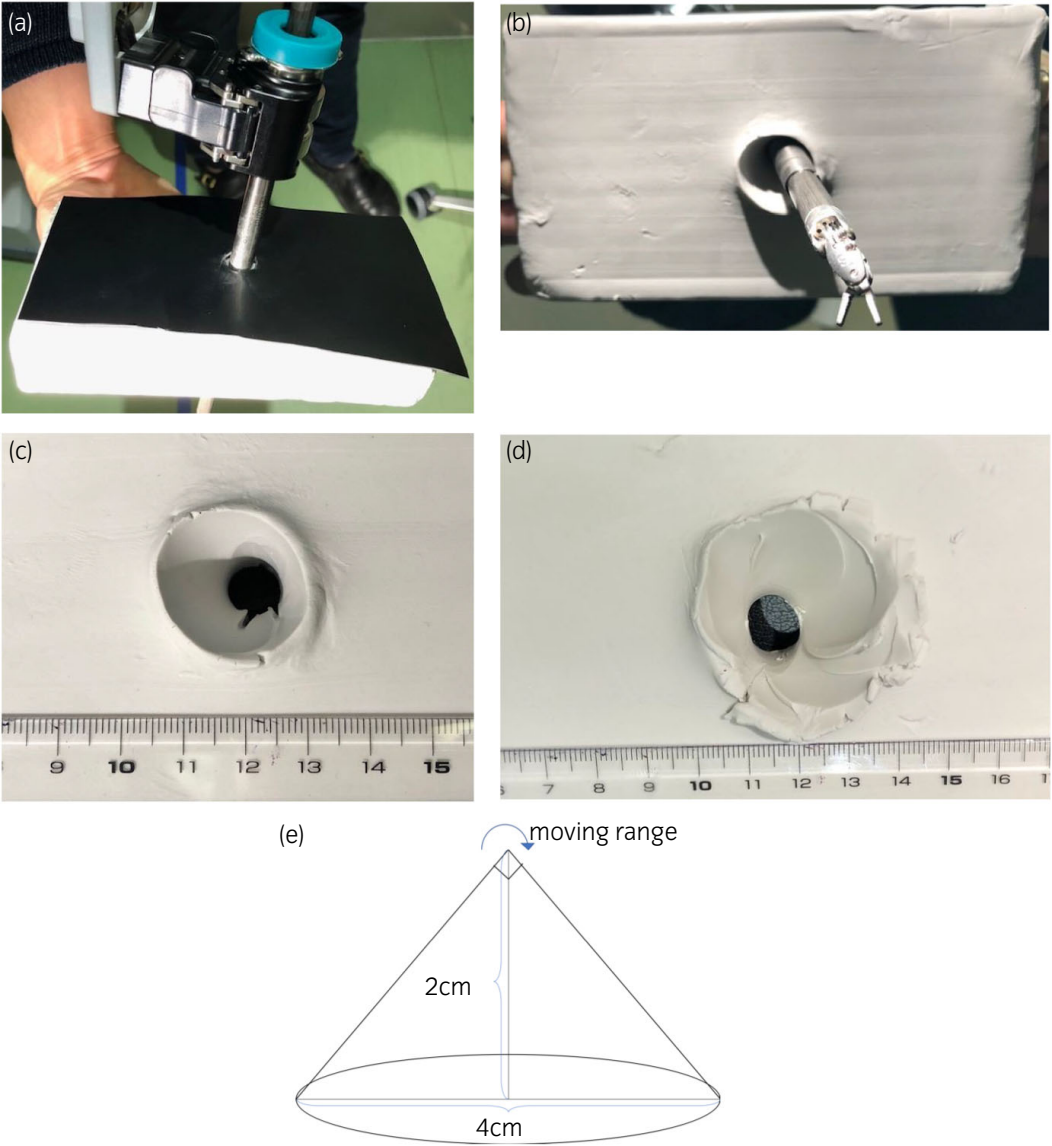
(a) A model that resembled clay as the abdominal wall and a rubber sheet as the skin. (b) The remote center was 2‐cm shallow. (c) The whole diameter when the remote center was correct position was 2.4 cm. (d) The whole diameter when the remote center was 2‐cm shallow was 4.5 cm. (e) The movable range of the robot’s arm when the remote center was 2‐cm shallow.

In robotic‐assisted surgery, the port is movable around a remote center, and it is set such that no extra external force is applied to the surgical wound regardless of the direction in which the arm moves. If the position of this remote center is shallow or deep, it may cause unexpected damage to the abdominal wall. To test this, we created a model that resembled clay as the abdominal wall and a rubber sheet as the skin, and used the da Vinci to experiment with how much damage to the abdominal wall would occur if the remote center was misaligned (Fig. 4a,b). When the remote center was correctly positioned, the abdominal wall actually had a 2.4‐cm hole, whereas when the remote center was 2‐cm shallow, the actual abdominal wall hole was 4.5 cm (Fig. 4c,d). Assuming that the movable range of the robot’s arm in the abdominal cavity is a cone with an apex angle of 90°, the diameter of the base is theoretically 4 cm when the height is 2 cm (Fig. 4e).

In this case, it is highly probable that the fascia at the port site was widened due to misplacement of the remote center, and, in addition, unfortunately the closure of the fascia was incomplete. Even if the fascia was severely damaged, hernia would not occur if the closure was sufficient. On the contrary, the hernia would not occur even if the fascia of the 8‐mm port without damage was insufficiently closed. Therefore, we must always confirm that the remote center positioned is located slightly above the parietal peritoneum when inserting the port. In the current case, although we confirmed that there was no displacement at the time of port insertion, we had the impression that one of the remote centers was positioned in a slightly shallower location at the time of port removal. In order to avoid the displacement of the port position during operations, reconfirming the base of the port and checking for any misalignment of the remote center when attaching the arm and setting the forceps in the pelvis is key. This is the first report to study the effects of port site hernia and misplacement of remote center.

In the current case, when the patient presented with abdominal symptoms, we immediately checked by contrast‐enhanced CT scan and detected a port site hernia relatively early, thus avoiding intestinal necrosis. When abnormal abdominal radiographs or symptoms occur after robot‐assisted surgery, it is important to investigate a possible incarceration of the intestinal tract at the port position through a contrast‐enhanced CT scan.

## Conflict of interest

The authors declare no conflict of interest.
